# Magnetic Resonance Imaging Study on Older Patients with Cognitive Impairment and Depression

**DOI:** 10.2174/0115734056281104241220113235

**Published:** 2025-01-02

**Authors:** Shuang Zhang, Yuping Qin, Meng Ding, Jining Yang, Tao Zhang

**Affiliations:** 1 School of Life Science and Technology, University of Electronic Science and Technology of China, Chengdu, Sichuan, China; 2 School of Artificial Intelligence, Neijiang Normal University, Neijiang, Sichuan, P.R. China; 3 High Field Magnetic Resonance Brain Imaging Laboratory of Sichuan, University of Electronic Science and Technology of China, Chengdu, Sichuan, China; 4 NJNU-OMNISKY Smart Medical Engineering Applications Joint Laboratory, Neijiang Normal University, Neijiang, Sichuan, P.R. China

**Keywords:** Depression, Older patients, Magnetic resonance imaging (MRI), Difference analysis, Correlation analysis of cognitive ability, Montreal cognitive assessment (MoCA)

## Abstract

**Background:**

Understanding brain changes in older patients with depression and their relationship with cognitive abilities may aid in the diagnosis of depression in this population. This study aimed to explore the association between brain lesions and cognitive performance in older patients with depression.

**Methods:**

We utilized magnetic resonance imaging data from a previous study, which included older adults with and without depression. Smoothed Regional Homogeneity (ReHo) and local brain Amplitude of Low-frequency Fluctuation (ALFF) values were assessed to examine brain activity.

**Results:**

The analysis revealed decreased ReHo in the left middle temporal gyrus, left middle frontal gyrus, and left precuneus, as well as increased local ALFF in the right middle occipital gyrus, left postcentral gyrus, and right precentral gyrus in older patients with depression. These alterations may contribute to behavioral and cognitive changes. However, no significant relationship was found between ReHo values and Montreal Cognitive Assessment (MoCA) scores. In contrast, increased local ALFF in the left postcentral gyrus and right precentral gyrus was negatively correlated with MoCA scores.

**Conclusion:**

This study demonstrated a significant association between regional brain alterations in patients with depression and cognitive behavior. Thus, this work identified functional brain regions and cognitive performance in older adults with depression, highlighting specific brain regions that play a crucial role in cognitive abilities in this population.

## 1 INTRODUCTION

Depression in older adults (aged ≥60 years) is often associated with symptoms commonly seen in this population, such as fatigue, physical discomfort, insomnia, appetite changes, and weight fluctuations [1]. These physical symptoms can be misinterpreted or overlooked due to aging, making depression more challenging to diagnose in older individuals [2]. Additionally, older adults face shrinking social circles, the loss of friends and family, and declining health, all of which contribute to feelings of loneliness and social isolation, factors that increase the risk of depression in this age group [3].

According to the World Health Organization (WHO), older adult patients with depression account for 3–5% of the global population and 7–10% of the global older adult population specifically [4, 5]. Depression is a significant factor in diminishing the quality of life for older adults. This condition manifests as a pervasive negative emotional state, characterized by low mood, crying, sadness, disappointment, decreased activity, slowed thinking, and cognitive decline [6]. Depression is often accompanied by anxiety, which can manifest as excessive worry, nervousness, self-doubt, and restlessness. Older adults with depression may also experience cognitive impairments, including difficulty concentrating, memory loss, and impaired decision-making. These cognitive issues can sometimes be misdiagnosed as Alzheimer's disease, but tend to improve with effective treatment of the depressive disorder [7, 8]. Therefore, analyzing cognitive abilities in older patients with depression is crucial.

In a previous study using Magnetic Resonance Imaging (MRI) to examine older patients with depression, resting-state functional MRI (rs-fMRI) and structural MRI (sMRI) data were systematically collected and compared with multimodal data, including clinical neurological assessments [9, 10]. Morphological analysis of brain gray matter revealed significant enlargement in regions, such as the left inferior temporal gyrus and the cortex surrounding the right calcarine fissure, along with reductions in areas, such as the left parahippocampal gyrus and left lentiform pallidum, in older adults with depression, compared to healthy controls. Amplitude of Low-frequency Fluctuation (ALFF) analysis revealed elevated local brain activity in the left postcentral gyrus and right precentral gyrus of the depression group, suggesting that older adults with depression exhibit significant metabolic changes and increased activity in specific brain regions [11-17]. Notably, the intensity of brain activity in the superior occipital gyrus was positively correlated with Hamilton Depression Rating Scale (HAMD) scores [18].

While these findings have advanced our understanding of brain changes in older patients with depression, the relationship between local brain ALFF and cognitive ability in this population has not been thoroughly explored. Cognitive function plays a crucial role in the diagnosis and treatment of depression, and its evaluation is critical for improving outcomes.

This study aimed to analyze differences in resting-state activity between older adults with and without depression and investigate the correlation between these changes and cognitive ability, as assessed by the Montreal Cognitive Assessment (MoCA) scale. By combining functional, structural, and clinical data, we sought to provide insights into the brain changes associated with depression in older adults and offer a theoretical basis for establishing diagnostic criteria and evaluating the effectiveness of antidepressant treatments in this population.

## 2. METHODS

### 2.1 Data Source

The data used in this study were previously utilized in an earlier investigation [18].

#### 2.1.1 Older Adults with Depression (Study Group)

##### 2.1.1.1 Inclusion Criteria

(1) Meeting DSM-V diagnostic criteria for depression; (2) Aged 50–80 years and of Han ethnicity, regardless of sex; (3) No pre-existing neurocognitive impairment, disorder, or related diseases; no serious concomitant somatic diseases; no history of drug or alcohol dependence. Post-stroke depression must be excluded in the absence of any nervous system disease; (4) Visual acuity, hearing, and limb function sufficient to complete neuropsychological assessments; ability to undergo MRI with no significant organic brain changes; (5)The patient and/or legal guardian must be able to read, understand, and sign the informed consent form, and be willing to cooperate and comply with study requirements.

##### 2.1.1.2 Exclusion Criteria

(1) Presence of severe physiological, infectious, or immune system diseases; (2) Contraindications for MRI, including metal implants, large tattoos, claustrophobia, metal-filled porcelain teeth, or metal braces/dentures that cannot be removed during scanning; (3) Significant symptom fluctuations within 2 weeks; (4) Moderate (difficulty hearing at close range) or severe deafness; (5) Suicide attempts or serious suicidal tendencies within the last 12 months, a score of ≥8 on the suicide risk assessment, or a score of >6 on the suicidal ideation scale during risk assessment; (6) Clinically significant abnormalities in laboratory tests (blood, urine, liver, kidney functions, etc.) affecting safety or requiring special treatment was required; and (7) Refusal by patient or legal guardian to sign the informed consent form.

#### 2.1.2 Control Group

##### 2.1.2.1 Inclusion Criteria

(1) No pre-existing neurocognitive impairment, disorder, or related diseases; (2) Aged 50–80 years and of Han ethnicity, regardless of sex; (3) No pre-existing neurocognitive impairment, disorder, or related diseases; no serious concomitant somatic disease; no history of drug or alcohol dependence. Post-stroke depression must be excluded in the absence of any nervous system disease (same as item (3) above); (4) Visual acuity, hearing, and limb function sufficient to complete neuropsychological assessments; able to complete MRI with no significant organic brain changes; and (5) The participant and/or legal guardian must be able to read, understand, and sign the informed consent form, and be willing to cooperate and comply with study requirements.

##### 2.1.2.2 Exclusion Criteria

(1) Presence of depression, neurocognitive impairment, disorder, or related diseases; (2) Contraindications for MRI, including metal implants, large tattoos, claustrophobia, metal-filled porcelain teeth, or metal braces/dentures that cannot be removed during scanning; (3) Presence of serious physiological, infectious, or immune system diseases; (4) Moderate difficulty hearing at close range or severe deafness; (5) Suicide attempts or serious suicidal tendencies within the last 12 months, a score of ≥8 on the suicide risk assessment, or a score of >6 on the suicidal ideation scale during risk assessment; (6) Refusal by the participant or legal guardian to sign the informed consent form.

#### 2.1.3 Exit Criteria (Same Criteria Apply to both the Older Adult Depression and Normal Control Groups)

(1) Inability to undergo MRI; (2) Sudden onset of serious systemic disease; (3) Serious adverse events; (4) Suicide risk of ≥8 or a total score of >6 on the suicide ideation scale; and (5) Revocation of informed consent by the volunteer, family members, guardians, or legal representatives.

#### 2.1.4 Collection of Demographic Data

(1) Basic participant information: name, age, sex, ethnicity, handedness, marital status, education level, living arrangements, family relationships, occupation, smoking status, and drinking status.

(2) Medical history in the older adults with depression group: age at first onset, previous hospitalizations, previous drug treatments (dosage, duration, and effects), and presence of other diseases.

#### 2.1.5 Data Collection Environment and Parameters: Preparation

Imaging data were collected using a 3.0-T MRI system (GE Healthcare, Chicago, IL). Participants were instructed to arrive at the preparation room 30 min prior to scanning for a briefing on safety precautions and to be familiar with the experimental environment, thereby minimizing psychological fluctuations. Informed consent for the study and MRI procedure was obtained. Before scanning, participants changed into appropriate attire, wore noise-cancelling earplugs, and were scanned with a metal detector to ensure no metal objects entered the MRI room. During scanning, participants lay flat on the MRI table, were instructed to keep their heads still, avoid distracting thoughts, and remain awake.

#### 2.1.6 Data Collection Environment and Parameters: MRI Experimental Procedures

The functional imaging parameters were as follows: repetition time (TR) = 2000 ms; echo time (TE) = 30 ms; slice thickness = 4 mm; field of view (FOV) = 24 cm × 24 cm; flip angle (FA) = 90°; and matrix size = 64 × 64. The structural imaging parameters were: resolution = 1 mm × 1 mm; slice thickness = 1 mm; inversion time (T1) = 450 ms; FOV = 25.6 cm × 25.6 cm; and matrix size = 256 × 256.﻿

### 2.2 Difference Analysis

To analyze differences between the smoothened regional homogeneity (SmReHo) and the local brain ALFF in the two imaging datasets, a difference analysis was performed comparing SmReHo and ALFF values.

### 2.3 Inter-group ReHo difference Analysis based on Resting-state

ReHo measures the homogeneity of temporal signals across a voxel and its neighboring voxels using the Kendall coefficient of concordance (KCC) [12]. Since two-sample t-test analyses require normal distribution assumptions, and ReHo values often display poor normality, the smoothed mReHo and zReHo values, which more closely follow a normal distribution, were used for analysis. SmReHo was therefore chosen for the voxel correlation analysis in this study (s indicates smoothing of mReHo).

Two-sample t-tests were conducted using the “dpabi” software package in MATLAB 2017b (MathWorks, Natick, MA). Voxel *p*-values (<0.005), cluster *p*-values (<0.05), and cluster sizes (>27) were corrected using the Gaussian random field (GRF) method, and the results were visualized using the dpabi Viewer and BrainNet Viewer.

### 2.4 Inter-group fALFF Analysis based on Rresting-state

ALFF measured the amplitude of low-frequency oscillations, calculated through Fourier transformation to assess hemodynamic signal fluctuations in a specific frequency range (0.01–0.08 Hz). This provides insight into the intensity of local brain activity. Since two-sample t-tests were used, and ALFF values typically poor normality, we selected the fractional ALFF (fALFF), which normalizes the data by dividing ALFF by the total amplitude across the frequency spectrum. The distribution of mALFF and zALFF values was consistent with normality, and zfALFF was chosen for parameter and correlation analysis. Two-sample t-tests were conducted using the dpabi software package in MATLAB 2017b. The voxel *p*-value threshold was set at <0.001, the cluster *p*-value threshold at <0.05, and the cluster size at >27 voxels. GRF-correction was applied to the results, which were visualized using the dpabi Viewer and BrainNet Viewer.

### 2.5 Cognitive Ability Correlation Study

To assess the cognitive abilities of older patients with depression, we conducted a correlation analysis between ALFF and ReHo values and MoCA scores [19,20]. A previous comparison found no significant relationship between ReHo values and MoCA scores [18], so the focus on the current analysis was on the correlation between ALFF values and MoCA scores.

Correlation analyses were performed using the statistical functions in dpabi. Local brain ALFF values in the older depression and control groups were correlated with MoCA scores. GRF correction was applied to voxel *p*-values (<0.005), cluster *p*-values (<0.05), and cluster sizes (>27), and the results were visualized using the dpabi Viewer and BrainNet Viewer.

## 3 RESULTS

### 3.1 Patient Characteristics

The characteristics of the study participants are presented in Table **1**. As a detailed analysis of these data has been reported previously [18], we have provided only demographic and clinical information here. Statistical analysis of basic information from both groups, using SPSS 22.0 (IBM, Armonk, NY), revealed no significant differences in sex, age, or years of education between the two groups (p>0.05) (Table **1**).

### 3.2 Inter-group ReHo Difference Analysis based on Resting-state

The results of inter-group ReHo difference analysis based on resting-state are shown in Table **2** and Fig. (**1**). The two-sample t-test comparison of SmReHo between the older depression group and the control group revealed regions with reduced ReHo in the left middle temporal gyrus, left middle frontal gyrus, and left precuneus in the depression group.

### 3.3 Inter-group fALFF Difference Analysis based on Resting State

The detailed results of the inter-group fALFF difference analysis based on resting-state are shown in Table **3** and Figs. (**2** and **3**). The two-sample t-test of fALFF values between the older depression group and control group identified regions of elevated local brain ALFF in the left postcentral gyrus and the right precentral gyrus in the depression group.

### 3.4 Cognitive Ability Correlation Study

**Fig. (4):**
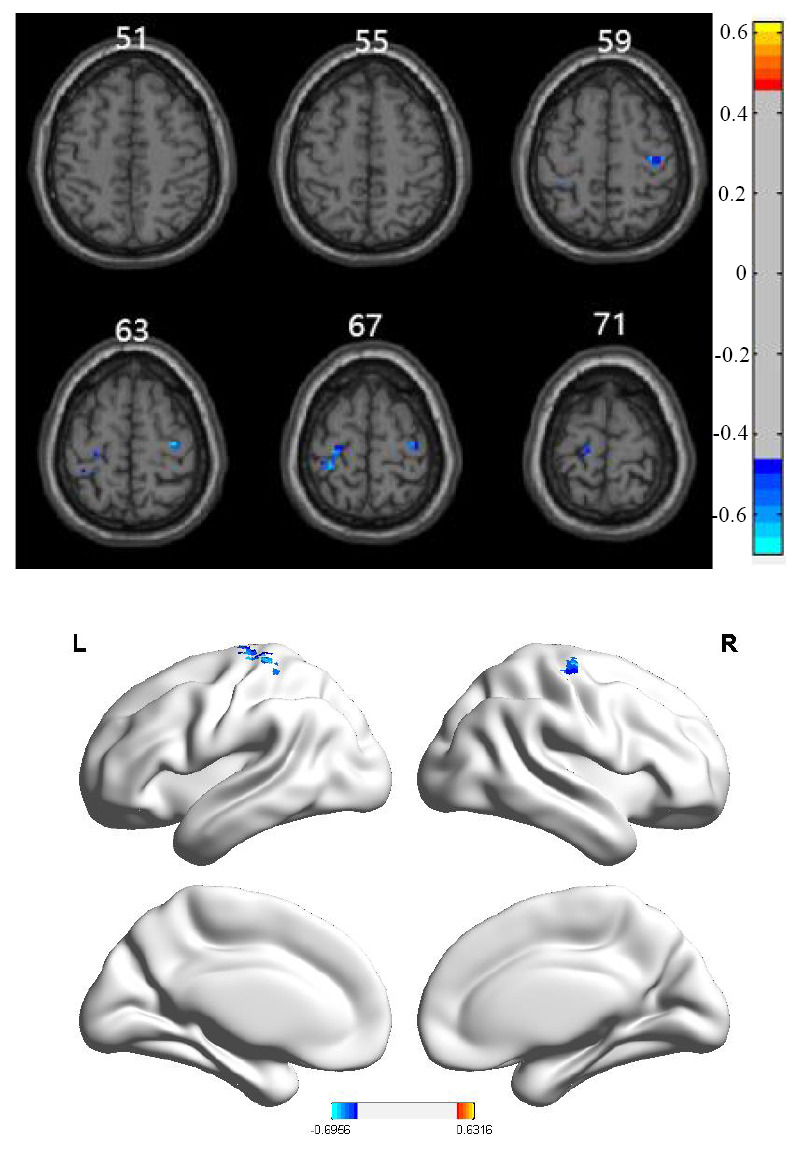
Results of correlation analysis between subjects’ local brain activity intensity (ALFF) and Montreal Cognitive Assessment (MoCA) scale scores. The voxel *p*-values (< 0.005) and cluster *p*-value (< 0.05) were corrected using GRF correction. The image displays 2D scans (left) and 3D scans (right) of the cortex. The cool-colored regions indicate where ALFF values negatively correlated with MoCA scores.

The correlation analysis between ALFF values and MoCA scores in both the older depression and control groups (Table **4** and Fig. **4**) showed a negative correlation between ALFF values and MoCA scores in the left postcentral gyrus and right precentral gyrus.

## 4 DISCUSSION

This study identified regions with reduced SmReHo in older patients with depression, specifically in the left middle temporal gyrus, left middle frontal gyrus, and left precuneus. These RehO differences were primarily concentrated in the temporal and parietal lobes, compared to the control group. Damage to the temporal lobe can result in activity disorders, such as epileptic seizures, and impairments in memory, hearing, speech, and vision. In contrast, regions with elevated local ALFF in the older depression group, compared to the control group, included the right middle occipital gyrus, left postcentral gyrus, and right precentral gyrus. The abnormal brain regions identified through zfALFF analysis were predominantly located in the frontal, parietal, and occipital lobes, consistent with previous studies. Lesions in the frontal lobe may impair motor activity, speech, writing, and other neurological functions.

Additionally, we observed a significant reduction in the gray matter volume of the parahippocampal gyrus in older patients with depression (Table **5**). Although no changes were observed in the gray matter volume of the hippocampus, the parahippocampal gyrus, hippocampus, corpus albicans, anterior thalamic nuclei, and cingulate gyrus form the hippocampal loop, a structure involved in higher neural functions, such as memory and emotional regulation. Abnormalities in the parahippocampal gyrus may be indicative of underlying conditions, such as schizophrenia, Alzheimer’s disease, or depressive disorder. Therefore, depression in older adults requires further investigation to understand its broader implications.

## CONCLUSION

In this study, SmReHo and local ALFF assessments in brain MRI scans of older adults with depression and healthy controls revealed reduced ReHo in the left middle temporal gyrus, left middle frontal gyrus, and left precuneus regions, as well as elevated ALFF in the right middle occipital, left postcentral gyrus, and right precentral gyrus regions. Damage to these brain regions may lead to behavioral and cognitive changes in older adults. While no significant correlation was found between ReHo values and MoCA scores, a negative correlation was observed between ALFF and MoCA scores in the left postcentral and right precentral gyri. This study contributes valuable data on the functional brain regions associated with cognitive abilities in older adults with depression.

## Figures and Tables

**Fig. (1):**
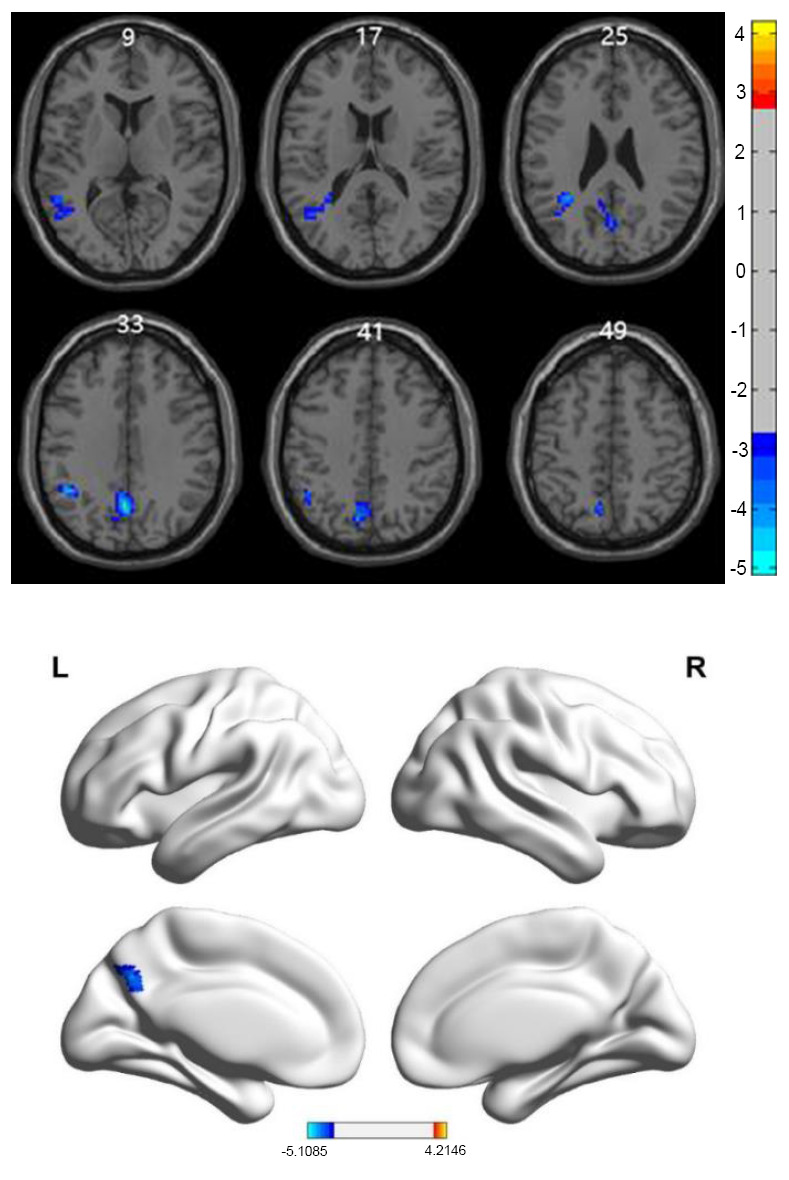
Two-sample *t*-test results of SmReHo between the study and control groups based on resting-state fMRI. Thresholds for the voxel *p*-value (<0.005) and cluster *p*-value (<0.05) were corrected using the GRF method. The left side of the figure shows 2D scans and the right side shows 3D scans of the cortex. Cool colors in the images indicate regions where the SmReHo values in the study group were lower than those in the control group. SmReHo, smoothened regional homogeneity; fMRI, functional magnetic resonance imaging; GRF, Gaussian random field.

**Fig. (2):**
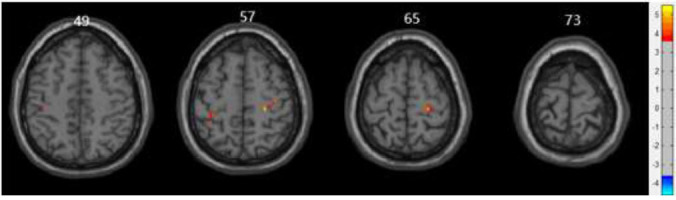
Imaging results of the two-sample *t*-test of zfALFF based on resting-state fMRI. The image data were corrected using GRF correction (voxel *p*-values < 0.001, cluster *p*-value < 0.05). Warm-colored regions in the image represent areas where the zfALFF values in the study group were greater than those in the control group. zfALFF, Z-standardized fractional amplitude of low-frequency fluctuation; fMRI, functional magnetic resonance imaging; GRF, Gaussian random field.

**Fig. (3):**
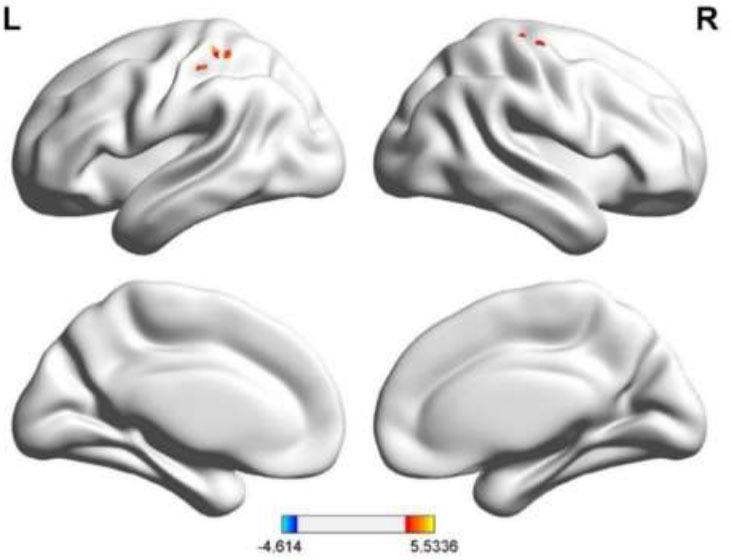
Three-dimensional cortical projection results of the two-sample t-test of zfALFF between both groups. The results were corrected using GRF correction (voxel *p*-values < 0.001, cluster *p*-value < 0.05). In the 3D cortical projection, warm-colored regions indicate areas where the zfALFF values in the study group were greater than those in the control group. zfALFF, Z-standardized fractional amplitude of low-frequency fluctuation; GRF, Gaussian random field.

**Table 1:** Demographics and clinical scale results for the Late-life Depression (LLD) and Healthy Control (HC) groups.

-	LLD Group	HC Group	*t*-value	*p*-value
Number of participants	13	24	-	-
Sex	9 women, 4 men	11 women, 13 men	-	0.300
Age (years)	62.46±8.42	57.13±7.24	2.022	0.051
Education (years)	9.46±4.82	7.96±3.70	1.059	0.297

**Notes:** Data are presented as mean ± standard deviation. The *p*-value for sex was obtained using the chi-square test, while *p*-values for other variables were calculated using two-sample *t*-tests.

**Table 2:** Two-sample *t*-test results of SmReHo between the study and control groups based on resting-state fMRI.

Group	Encephalic Region (AAL)	MNI coordinates at the Peak Point			Continuous Voxel Value	*t-*value at the Peak Point
		x	y	z		-
Study group < control group	Left middle temporal gyrus	–45	–51	33	76	–4.702
	Left middle frontal gyrus	–24	33	15	38	–4.616
	Left precuneus	–3	–63	36	154	–5.1085

**Notes:** SmReHo, smoothened regional homogeneity; MNI, Montreal Neurological Institute; fMRI, functional magnetic resonance imaging; AAL, automatic anatomical labeling.

**Table 3:** Two-sample *t*-test results of zfALFF between the study and control groups based on resting-state fMRI.

Group	Encephalic Region (AAL)	MNI Coordinates at the Peak Point			Continuous Voxel Value	*t*-value at the Peak Point
		x	y	z		-
Study group < control group	Left postcentral gyrus	–36	–39	60	16	5.422
	Right precentral gyrus	21	–27	60	15	5.534
	Right middle occipital gyrus	–10	–33	60	13	5.387

**Notes:** zfALFF, Z-standardized fractional amplitude of low-frequency fluctuation; MNI, Montreal Neurological Institute; fMRI, functional magnetic resonance imaging; AAL, automatic anatomical labeling.

**Table 4:** Results of correlation analysis between patients’ local brain activity intensity and montreal cognitive assessment scores.

Group	Brain Region (AAL)	MNI Coordinates at the Peak Point			Continuous Voxel Value	*t*-value at the Peak Point
		x	y	z		
Negatively correlated	Left postcentral gyrus	–27	–24	66	20	–0.649
	Right precentral gyrus	33	–18	63	19	–0.696

**Notes:** AAL, automatic anatomical labeling; MNI, Montreal Neurological Institute.

**Table 5:** Two-sample t-test results of gray matter volume in the study and control groups based on VBM analysis.

Group	Brain Region (AAL)	MNI Coordinate of Peak Point			Continuum Voxel Value	t-value at Peak Point
		x	y	z		
Study group > control group	Left inferior temporal gyrus	−45	−15	−19.5	133	8.255
	Cortex around the right talus	31.5	−6	0	233	7.925
	Left superior occipital gyrus	−4.5	−103.5	10.5	92	6.644
	Right inferior frontal gyrus (triangular part)	39	28.5	16.5	114	6.195
Study group > control group	Left middle frontal gyrus	−37.5	55.5	22.5	321	7.568
	Right angular gyrus	55.5	−34.5	58.5	207	6.364
Study group < control group	Left parahippocampal gyrus	1.5	−7.5	−18	62	−5.281
	Left lenticular globus pallidus	−24	−12	3	146	−4.925
	Left caudate nucleus	−7.5	−6	19.5	159	−4.980
	Right caudate nucleus	6	4.5	13.5	156	−5.081
	Right supplementary motor area	−1.5	−13.5	78	143	−4.917
	Left paracentral lobule	−3	−14	66	136	−4.625

**Note: **VBM, voxel-based morphometry; AAL, automatic anatomical labeling; MNI, Montreal Neurological Institute.

## Data Availability

The datasets used in this study will be available upon request from the corresponding authors [S.Z] and [T.Z]. The data are not publicly available due to privacy reasons.

## LIST OF ABBREVIATIONS

HAMD: Hamilton Depression Rating Scale
SmReHo: Smoothened regional homogeneity
ReHo: Regional homogeneity
zReHo: Z-transformed regional homogeneity
ALFF: Amplitude of low-frequency fluctuation
MoCA: Montreal Cognitive Assessment
WHO: World Health Organization
rs-fMRI: Resting-state functional MRI
sMRI: Structural MRI
HC: Healthy control
LLD: Late-life depression
zfALFF: Z-standardized fractional amplitude of low-frequency fluctuation
mALFF: Mean amplitude of low-frequency fluctuation
zALFF: Z-standardized amplitude of low-frequency fluctuation
fALFF: Fractional amplitude of low-frequency fluctuation

## STANDARDS OF REPORTING

STROBE guidelines were followed.
